# Site-Specific Gut Microbial Signatures in Non-Celiac Gluten Sensitivity

**DOI:** 10.1080/29933935.2024.2438621

**Published:** 2024-12-18

**Authors:** Kunal Dixit, Anam Ahmed, Alka Singh, Mitali Inamdar, Sonal Chavan, Rahul Bodkhe, Wajiha Mehtab, Ashish Chauhan, Sunil D. Saroj, Vineet Ahuja, Yogesh Shouche, Dhiraj Dhotre, Govind Makharia

**Affiliations:** a Symbiosis School of Biological Sciences, Symbiosis International (Deemed University), Pune, India; b Department of Gastroenterology and Human Nutrition, All India Institute of Medical Sciences, New Delhi, India; c National Centre for Cell Science, Pune, India; d Department of Integrative Biology, University of California, Berkeley, USA; e Department of Home Science, University of Delhi, New Delhi, India; f SKAN Research Trust, Bengaluru, India

**Keywords:** Gluten-free diet, irritable bowel syndrome, 16S rRNA amplicon library, shotgun metagenome, small intestine, human microbiome, microbial signature, gluten sensitivity

## Abstract

Gut microbiota in non-celiac gluten sensitivity (NCGS) has been poorly studied for its involvement in the disorder and site specificity. We investigated small intestinal, large intestinal and stool microbiota profiles in patients with NCGS and highly overlapping disorder irritable bowel syndrome (IBS) as well as effect of gluten-free diet (GFD) on microbiota in patients with NCGS. True NCGS patients were recruited based on serological response for anti-gliadin antibodies, 6-week gluten free diet (GFD) and symptom recurrence with gluten-rechallenge. Analyses using 16S rRNA gene amplicon and shotgun sequencing revealed community differences in core microbiome and diversity measures across sample types indicating dysbiosis mainly in mucosa-associated small intestinal microbiome of NCGS patients. Genera *Elusimicrobiaum, Succinivibrio*, *Bacillus* and *Alcaligenes* appeared as signatures in small intestine and stool in NCGS patients. Presence of differential taxa co-occurring at sampling sites, enabled recognition of site-specific microbial signatures. GFD led to a shift in mucosa-associated small intestinal core microbiome. Metagenome analysis revealed subtle differences in pathways for amino acid biosynthesis including L-ornithine. Mucosa-associated small intestine microbial structure was quite distinct in patients with NCGS in comparison to that with IBS.

## Introduction

Significant advancements in the knowledge about the spectrum of gluten-related disorders have widened our understanding of their pathophysiology. Non-celiac gluten sensitivity (NCGS) is gaining focus in recent years and is clinically present with plethora of both gastrointestinal symptoms such as abdominal pain, altered stool frequency, and extraintestinal symptoms such as nausea, headache, brain fog, tingling and numbness in hands and feet, fatigue, and musculoskeletal pain.^1,2^ NCGS is usually characterized by appearance of the above-mentioned symptoms after dietary intake of gluten and exclusion of both celiac disease (CeD) and wheat sensitivity diagnosis.^3^ Although no reliable data on the population-based prevalence are available, hospital-based study suggests a prevalence of 0.6–10.6% and this wide range of prevalence is mainly because of varying definitions of NCGS used in various studies.^4^

While NCGS is caused by ingestion of wheat, the exact downstream pathophysiology is still not well established.^5,6^ The proposed pathogenetic mechanisms include stimulation of innate immune response to gluten peptides or amylase-trypsin inhibitors, FODMAP effects and gut microbial factors.^7^ It is appropriate to hypothesize that microbial peptidases may have a role in modulating degradation of proteins from wheat by partial or complete digestion of immunogenic peptides^8^ resulting in either protection from the disorder or probable induction of the disorder. As NCGS is a fairly new diagnosis, only a few studies have investigated gut microbiota in these patients. Mazcorro et al. reported a significant increase in family *Ruminococcaceae* in NCGS patients when compared with healthy controls. Also, an increase in *Saccharibacteria, Actinobacillus* and *Finegoldia* in patients with NCGS and increased *Sphingobacterium* in healthy controls were found in the same study.^9^ In another study, an increase in *Peptostreptococcaceae* with a decrease in *Porphyromonadaceae* were reported in patients with NCGS, when compared to healthy controls.^10^

While studying microbiota in the stool is convenient, study of region-specific microbiota using comprehensive sampling from the human digestive system is likely to provide information that may be relevant in pathophysiology of NCGS.^11^ Differences in microbiome communities at different locations of the gastrointestinal tract has been reported in patients with disorders like CeD as well as colon polyps, highlighting importance of site-specific microbiome profiling in patients.^12,13^ One of the challenges for region-specific microbial study is to find healthy control subjects, especially sampling from the small intestine and large intestine, mainly due to ethical considerations.

Interestingly, symptoms of NCGS overlap with the symptoms of irritable bowel syndrome (IBS).^14^ However, no valid biomarker for NCGS diagnosis is available till date. Moreover, patients with IBS and positive Anti-gliadin antibodies (AGA) have been shown to respond to gluten-free diet (GFD) with reduction in gastrointestinal symptoms suggesting a possibility of NCGS being the real diagnosis in them.^15,16^ Lack of genetic and serological biomarkers as well as overlapping manifestation of IBS with NCGS, makes the diagnosis of NCGS further challenging.^17^

Using comprehensive sampling of the human gut, we thus planned to explore the microbial community structure of mucosa-associated small intestine, mucosa-associated large intestine and stool of patients with NCGS.^18^ Since obtaining biopsies from healthy controls is an ethical constraint, we recruited patients with AGA-negative IBS as disease controls and obtained the small intestinal biopsies, large intestinal biopsies and the stool samples from them. Therefore, we aimed at exploring the site-specific microbiome differences in patients with NCGS and AGA-negative IBS. We further unravel the modulation of site-specific microbiota after six weeks of treatment with GFD in patients with NCGS.

## Materials and methods

### Patient recruitment and sample collection

All patients with IBS were recruited based on Rome IV criteria and underwent routine screening tests such as stool microscopic examination (to rule out parasitic infections), stool occult blood test, as well as routine hematological tests and biochemical tests. The patients were further screened for CeD and gluten hypersensitivity using IgA Anti-tissue transglutaminase antibodies (antitTG) using Quanta Lite h-tTG IgA and AGA using Quanta Gliadin IgA and IgG, respectively. An AGA (IgA and/or IgG) titer of greater than 30 U/ml was considered positive for gluten sensitivity and patients were requested to undergo sigmoidoscopic examination for rectosigmoid biopsies and upper gastrointestinal endoscopic examination for collecting duodenal biopsies, which were used ahead for microbiome analysis. Stool samples were collected from patients prior (1 to 7 days) to sigmoidoscopic and endoscopic examination. IBS patients with AGA (IgA and/or IgG) titer of lower than 30 U/ml were considered negative for gluten sensitivity.

IBS patients recruited for the study were assessed for symptom severity using a five-question survey (IBS-SSS).^19^ IBS patients negative for gluten sensitivity were not put on GFD intervention. However, in case of IBS patients with elevated AGA levels (patients positive for gluten sensitivity), individual symptoms were assessed at baseline before starting GFD. These patients were assessed by the diagnostic questionnaire at day 0 to establish baseline symptoms using a self-administered instrument which consisted of a modified version of the Gastrointestinal Symptom Rating Scale (GSRS).^20^ Extraintestinal manifestations such as headache, brain fog, fatigue, numbness of limbs, joint pain, dermatitis, headache, and so on were also included in assessment. Top three symptoms were identified for every patient using a numerical rating scale for scores ranging from 1 (mild) to 10 (severe).^21^ GFD was started for these patients after consultation by a dietitian, and the symptoms were reassessed for improvement after 6 weeks. Responders were defined as patients who had at least 30% decrease in baseline symptom score in the top three symptoms as per the Salerno’s diagnostic criteria.^21^

Patients who responded to a GFD were then given a gluten rechallenge for 6 weeks, during which they were asked to restart their previous gluten-containing diets, and all symptoms were re-assessed. Stool samples and biopsies were collected after 6 weeks of the GFD (prior to gluten rechallenge) for further comparative analysis. Patients with recurrence or worsening of symptoms after restarting gluten were finally diagnosed as NCGS patients.^22^ Relatives of patients coming to the outpatient department were taken as healthy controls, and single-time point stool samples were collected from them for comparative analysis (*n* = 60).

Ethics approval was obtained from the Ethics Committees of both All India Institute of Medical Sciences (AIIMS), New Delhi (IEC/68/08.01.2016) and National Centre for Cell Science (NCCS), Pune (RP-58/2017). Informed and written consent was obtained from each participant of the study.

### DNA extraction, 16S rRNA, shotgun metagenome sequencing, and analyses

Stool microbial DNA extraction was performed using QIAamp 96 PowerFecal QIAcube HT Kit (QIAGEN: Valencia, CA, USA) with the help of QIAcube HT liquid handler system. DNeasy Blood and Tissue kit (QIAGEN, USA) was used for microbial DNA extraction from biopsy samples as per the manufacturer’s instructions.^23^ Purity and the concentration of DNA was checked using NanoDrop ND-1000 Spectrophotometer (NanoDrop Biotechnologies, USA). Extracted DNA was normalized to 100ng/µL and was stored at − 20°C until further use. V4 region of 16S rRNA gene was amplified with polymerase chain reaction using 515F (5′-GTGCCAGCMGCCGCGGTAA-3′)/806 R (5′- GGACTACHVGGGTWTCTAAT-3′) universal primer set with sequencing adapters (Integrated DNA Technologies), as described in the Earth Microbiome Project.^24^ Samples were further processed for 16S rRNA amplicon and shotgun sequencing. For 16S rRNA gene amplicon sequencing the amplified products were purified and pooled to equimolar concentrations (PicoGreen) and sequenced using Illumina MiSeq platform as per the manufacturer’s instructions (Illumina technologies, USA). Sequencing was performed using V4 paired-end (2 × 250 bp) chemistry.

### 16S rRNA gene sequencing and Statistics

Data for a total 107 stool samples and 95 mucosa samples were obtained. Total 111,830 ± 39249 reads per sample (80.50% non-chimeric) were obtained for stool samples; whereas 132,029 ± 52210 (82.96% non-chimeric) and 150,475 ± 91715 (88.23% non-chimeric) reads per sample were obtained for small and large intestinal samples, respectively. Analysis of 16S rRNA gene metabarcoding sequencing data was performed using DADA2 (v1.16) pipeline available at http://benjjneb.github.io/dada2/tutorial_1_6.html. Primer sequences were removed using trimLeft parameters (23 & 19 for forward and reverse reads respectively) in the filterAndTrim function of DADA2.^25^ Further, sequences with a Phred quality score below 30 (Q30) and short length (<220bp for R1 reads and < 190 bp for R2 reads) were removed. The reads were then denoised, merged and were checked for chimeric sequences using a consensus approach in DADA2. Taxonomy was assigned to the amplicon sequence variants (ASVs) using Ribosomal Database Project naive Bayesian classifier and non-redundant 16S rRNA gene database, SILVA (v138).^26^

The downstream analysis was performed using R (v4.1.1) (R Core Team 2020) with phyloseq, ggplot2, ggpubr, ape, vegan, and reshape2 packages. Alpha diversity indices such as Shannon index, Simpson’s index and observed ASVs were calculated (*p*-values <0.05) to compare the microbial community structure within study groups. Relative abundances of genera associated with study groups were calculated and compared to highlight the differences in community compositions. Moreover, to interpret the observed taxonomic variation pattern among study groups, non-parametric multidimensional scaling (NMDS) with significance testing by adonis2 from the vegan package was employed. Core microbiome analysis was performed using the microbiome package with considering taxa present in two thirds of samples (67% prevalence) as core microbiome. Differentially abundant taxa were calculated with the help of DESeq2 package in R.^27^ Co-occurrence and co-exclusion network analysis was performed for the taxa with relative abundance > 0.2% and > 0.05% for stool and biopsy samples, respectively. Negative interactions were filtered out of the networks to visualize mutualistic interactions. Also, nodes with degrees less than two were discarded to identify the cliques with three or more interactions. correlation was performed using the Hmisc package and matrix was visualized using igraph and viridis in R.

### Shotgun metagenome sequencing and statistics

Stool samples for NCGS (before and after GFD) and AGA-negative IBS patients were further processed for shotgun sequencing. Sequencing was outsourced to Medgenome labs, India. Metagenome sequencing libraries were prepared using KAPA DNA HyperPrep kit. In brief, the DNA was sheared using Covaris ultrasonicator and then subjected to a sequence of enzymatic steps for repairing the ends and tailing with dA-tail followed by ligation of indexed adapter sequences. These adapter ligated fragments were then cleaned up using SPRI beads (Beckman Coulter Lifesciences, USA). Next, the clean fragments were indexed using limited cycle PCR to enrich the adapter ligated molecules. Finally, the amplified products were purified and checked for quality and quantity before sequencing. Illumina HiSeqX platform was used for the paired end shotgun sequencing with 150 × 2 chemistry. Total 10 gigabytes and 70,178,058 ± 10629840 reads per sample were obtained. Approximately 2.7% of the total reads were lost after adapter trimming and 19.10% of the reads were assigned to human reads. The remaining reads were used for the downstream analysis. Read quality distribution was estimated using the FastQC tool and only high-quality reads were retained (Q ≥ 30). Trimming was performed with the help of the BBDuk tool (trimq = 15, minlength = 50 and minavgquality = 15). After quality filtering, forward and reverse reads were concatenated in Linux. Merged reads were then processed ahead with the open source HUMAnN 3.0 analysis pipeline for shotgun data analysis, to filter out host DNA (Human genome build 38) and further to obtain accurate profiling of the abundance of microbial metabolic pathways, pathway coverage and gene families. MetaPhlAn 3.0 was used for read based microbial taxonomic assignment.^28^ Differential analysis of the species and pathway abundance was performed using Analysis of variance calculation (ANOVA) and hits were filtered for an adjusted *p* value < 0.05. Additional filters were put on the shortlisted hits with Wilcoxon signed-rank test to estimate the significantly differential species and pathway abundances between NCGS and AGA-negative IBS as well as NCGS at baseline and NCGS patients after GFD.

## Results

### Patient description and demographics

**Figure 1.**
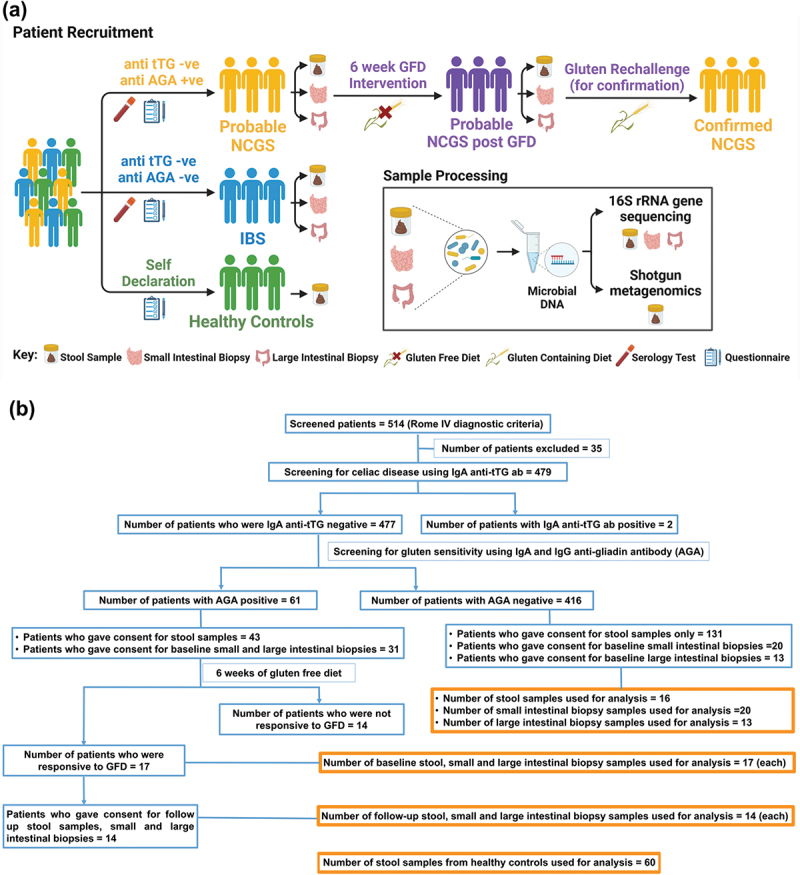
Study design. Patient recruitment and sample processing (a). Workflow for screening and sample collection (b).

To identify patients with NCGS, 514 adult patients with IBS were screened for participation in the study and 35 were excluded based on the inclusion and exclusion criteria. After the standard diagnostics, 31 patients with IBS (AGA positive) were put on GFD of which 17 were confirmed to have NCGS. Remaining 14 patients did not respond to the intervention. The current study is focused on reporting microbiome associated with true NCGS patients and how it is different with respect to AGA-negative IBS microbiome. The sample collection and patient recruitment scheme are presented in Figure 1a, b. Biological samples of 17 patients from each sampling site (small intestine, large intestine and stool samples) were taken ahead for microbiome profiling from NCGS patients before GFD. Whereas, 14 samples from each sampling site were collected and profiled after GFD. In case of AGA-negative IBS patients, 20 small intestinal biopsy samples, 13 large intestinal biopsy samples, and 16 stool samples were used for microbiome analysis.

The mean duration of symptoms was 4.8 ± 3.8 years. The mean IBS symptom severity score for NCGS patients at baseline and after 6 weeks of GFD was 194.7 ± 76.9 and 118.2 ± 59.6, respectively. The mean of top three symptoms (abdominal pain/discomfort, altered stool frequency, and altered stool consistency; max score = 30) for 17 NCGS patients at baseline and after 6 weeks of GFD was 20.9 ± 3.8 and 5.6 ± 2.6, respectively. Thus, the average percentage of improvement in symptoms of these patients was 72.7 ± 13.3. Detailed information regarding patient-wise demographic details, symptom score, response to GFD and diagnosis has already been published in our other article.^22^

### Microbial diversity differs in patients with NCGS in comparison to those with AGA-negative IBS

To map the microbiome in patients with NCGS patients and to explore the differences from the microbiota of patients with AGA-negative IBS, 16S rRNA gene amplicon profiling of the small intestinal mucosal biopsies, large intestinal biopsies and stool was performed in both the disorders.

#### Diversity in the small intestine mucosa-associated microbiota in patients with NCGS

**Figure 2.**
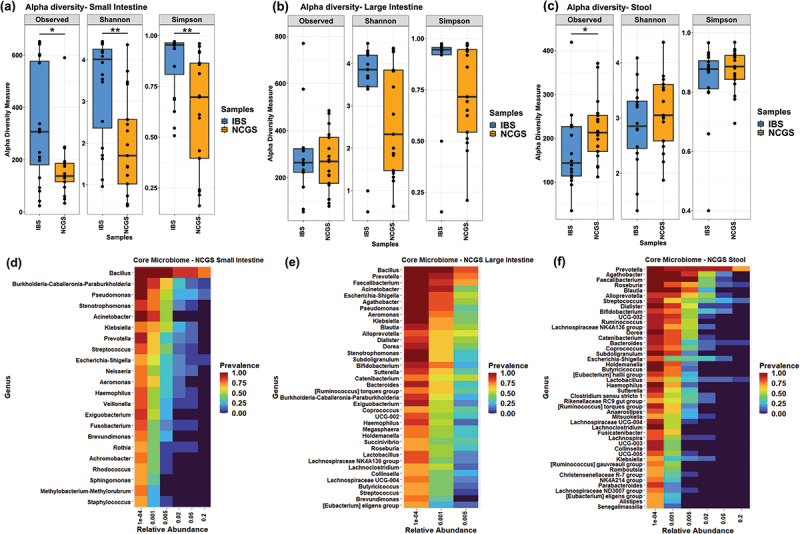
Bacterial diversity analysis. Alpha diversity of microbiota in patients with NCGS and AGA-negative IBS in the small intestine (a), large intestine (b) and stool samples (c). Core microbiome of patients with NCGS in the small intestine (d), large intestine (e) and stool samples (f).

There was a major difference in the small intestinal mucosa-associated microbial community structure in patients with NCGS and AGA-negative IBS. A significantly lower number of observed ASVs as well as bacterial evenness in the small intestinal mucosa of patients with NCGS was reported (*p* < 0.05; Wilcoxon signed-rank test) (Figure 2a). The small intestine mucosa associated microbiota of patients with NCGS had a dominance of *Bacillus* followed by *Actinobacillus, Burkholderia*, and *Escherichia/Shigella* and a lower abundance of potentially beneficial and previously reported SCFA-producing organisms such as *Faecalibacterium, Prevotella,* and *Veillonella* (Figure S1a).^29^ A high prevalence of *Bacillus, Burkholderia, Pseudomonas*, and *Klebsiella* and low prevalence of *Prevotella* indicate a possible dysbiosis in the small intestine of patients with NCGS. The core microbiome of small intestinal mucosa of patients with NCGS had a lower number of taxa in comparison to those in the large intestine and the stool (Figure 2d).

#### Diversity in the large intestine mucosa-associated microbiota in patients with NCGS

In case of large intestinal samples, similar number of ASVs were found in both NCGS and AGA-negative IBS patients. Whereas, NCGS patients showed a trend of lower Shannon and Simpson’s diversity indices when compared to AGA-negative IBS (Figure 2b). The mucosa-associated microbiota in the large intestine of patients with NCGS showed higher abundances of *Bacillus* and *Pseudomonas* and lower abundances of putative beneficial taxa such as *Faecalibacterium, Prevotella* and *Suterella*. On the other hand, mucosa-associated microbiota in the large intestine of patients with AGA-negative IBS had higher levels of *Bacteroides* and *Escherichia-Shigella* (Figure S1b). Further, *Bacillus* was observed as the most prevalent core organism followed by *Prevotella*, *Faecalibacterium* and *Acinetobacter* in the large intestine of patients with NCGS. Interestingly, none of these organisms were present as a part of core taxa for more than 0.5% relative abundance threshold indicating a high interindividual variation in the microbiome in terms of core taxa in these patients (Figure 2e).

#### Diversity in the stool-associated microbiota in patients with NCGS

The microbiota in the stool of NCGS patients showed the opposite trend as compared to that observed in the small and large intestinal microbiota. Higher bacterial richness and evenness was observed in NCGS patients based on alpha diversity measures; wherein only observed ASV count was significantly different (*p* < 0.05) (Figure 2c). While the stool samples of patients with NCGS had higher abundances of *Roseburia, Succnivibrio* and *Treponema* (Figure S1c), AGA-negative IBS patients exhibited higher abundances of *Bacillus, Bacteroides, Lactobacillus, Megamonas,* and *Megasphaera*. Additionally, patients with NCGS had *Prevotella* as a highest prevailing and abundant taxon in the stool. Moreover, taxa like *Faecalibacterium, Blautia, Bifidobacterium, Lactobacillus, Clostridia*, and so on, which are important for gut homeostasis and short chain fatty acid production were also present as core microbiome in the stool of patients with NCGS (Figure 2f).

Moreover, upon comparison of the stool microbiota of patients with NCGS to healthy controls, no significant differences in alpha diversity measures, dominant taxa, and core microbiome were observed (Figure S2a, c, d). Interestingly, scattered NCGS stool samples over the plot indicated higher interindividual variation in these patients in comparison to tightly clustered healthy individuals (Figure S2b).

Limited differences were observed between core microbiome of AGA-negative IBS and NCGS. Large intestinal microbiome in AGA-negative IBS patients found to have higher number of core taxa (Figure S3a-c). *Prevotella* was found prominently throughout the gut of AGA-negative IBS patients. Additionally, small intestinal samples of AGA-negative IBS patients were found to be dominated by taxa such as *Bacillus*, *Pseudomonas*, *Stenotrophomonas*. Whereas, *Faecalibacterium* was found as an abundant taxon in large intestine and stool samples along with *Escherichia-shigella*, *Dialister* as well as *Agathobacter*, *Bifidobacterium* respectively.

Additionally, microbiota associated with non-responders (AGA positive IBS patients) was compared to NCGS and AGA-negative IBS patients. Non-responders were found to have significantly higher alpha diversity (Observed ASV, Shannon index; *p* < 0.05) than NCGS patients (responders) in small intestine. In case of stool samples significantly lower number of observed ASVs were reported in non-responders as compared to responders to GFD. However, microbial diversity differences between non-responders and AGA-negative IBS patients were non-significant (Figure S4).

### Gut microbiota in patients with NCGS and AGA-negative IBS are site-specific

**Figure 3.**
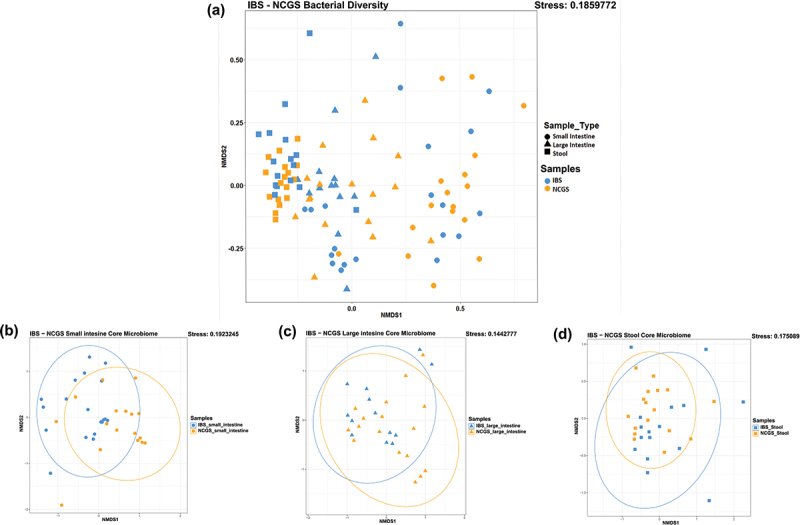
Showing site specificity of microbiome; NMDS analysis based of sampling loci between patients with NCGS and AGA-negative IBS (a). NMDS analysis for the core microbiome of the small intestine (b), large intestine (c) and stool samples (d).

Non-metric multidimensional scaling (NMDS) revealed a distinct variation in the microbial community structure in three different sampling locations, namely small intestine, large intestine and stool. The samples appear as a spectrum with different clusters on the ordination plot (Figure 3a). This site-wise distribution of samples was significantly different for all three sampling sites for both NCGS and AGA-negative IBS (Adonis, *p* < 0.05) revealing that the microbiome in these patients were highly site-specific. Further analyses revealed that, between the study groups, microbiota was significantly different only in the small intestinal biopsies. This might suggest that manifestations of NGCS could be due to altered microbial communities in the small intestine. Furthermore, a distinct clustering of core microbiota of patients with NCGS and AGA-negative IBS was observed in small intestinal mucosa biopsies and the stool (Adonis, *p* < 0.05), no such difference was found in the core microbiome of large intestinal mucosa (Figure 3b, c, d).

### Site-specific co-occurrence reveals differential microbial signatures of NCGS

**Figure 4.**
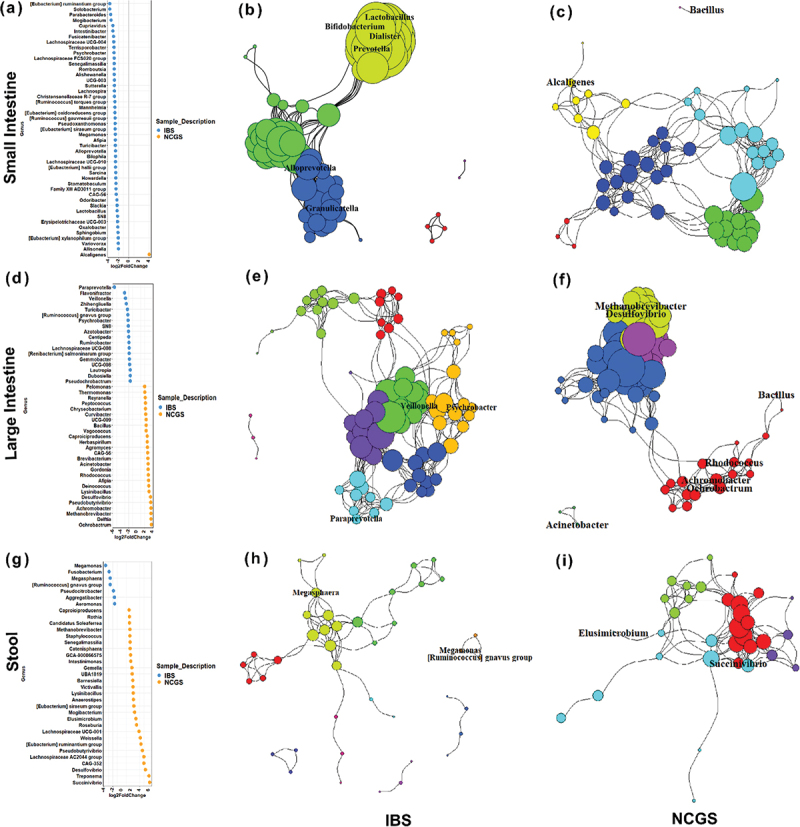
Signature taxa in different sample types. Differentially abundant taxa between patients with AGA-negative IBS and NCGS in the small intestine (*p* < 0.01) (a), large intestine (d) and stool samples (g). Co-occurrence networks with signature taxa in patients with AGA-negative IBS (b, e, h) and NCGS (c, f, i).

The site-wise microbiome differences mapped were further tested for determining microbial signatures between patients with NCGS and AGA-negative IBS based on differential abundance and bacterial co-occurrence-based network analysis. Differential abundance analysis using DESeq2 revealed presence of bacterial taxa in significantly different amounts between the two groups whereas, co-occurrence networks showed the bacterial genera interacting with each other based on a positive correlation. We observed 47 significantly different taxa (*adj*. *p* < 0.01) in the small intestinal mucosa (Figure 4a); and 44 within the large intestinal mucosal biopsies and 35 differential taxa (*adj*. *p* < 0.05) (Figure 4d) in stool samples (Figure 4g), respectively of patients with NCGS and AGA-negative IBS.

The differential taxa were further mapped onto bacterial co-occurrence networks resulting in the site-specific signatures, i.e., taxa that were both significantly different and form key nodes of the network in patients with NCGS or AGA-negative IBS. Higher network modularity (0.563), diameter (3.541) and lower density (0.177) suggested a higher dysbiosis in the small intestine of patients with NCGS compared to that in AGA-negative IBS [supplementary Data 1]. Small intestinal mucosa, which showed the most differences in microbiota structure, displayed *Bacillus* and *Alcaligenes* as signatures in NCGS. In the small intestine of AGA-negative IBS patients, *Lactobacillus, Bifidobacterium, Dialister, Prevotella, Alloprevotella, Granulicatella* demonstrated prominence as signature taxa (Figure 4b, c). In the large intestine, while *Methanobrevibacter, Desulfovibrio, Bacillus, Rhodococcus, Achromobacter, Ochrobactrum, Acinetobacter* were identified as signature taxa in NCGS patients; *Veillonella, Psychrobacter, Paraprevotella* however emerged as signature taxa in patients with AGA-negative IBS (Figure 4e, f). In the stool samples, patients with NCGS exhibited *Elusimicrobium, and Succinivibrio* as signature taxa; AGA-negative IBS patients were observed to have a clique involving *Megasphaera, Megamonas, [Ruminococcus] gnavus* (Figure 4h, i).

**Figure 5.**
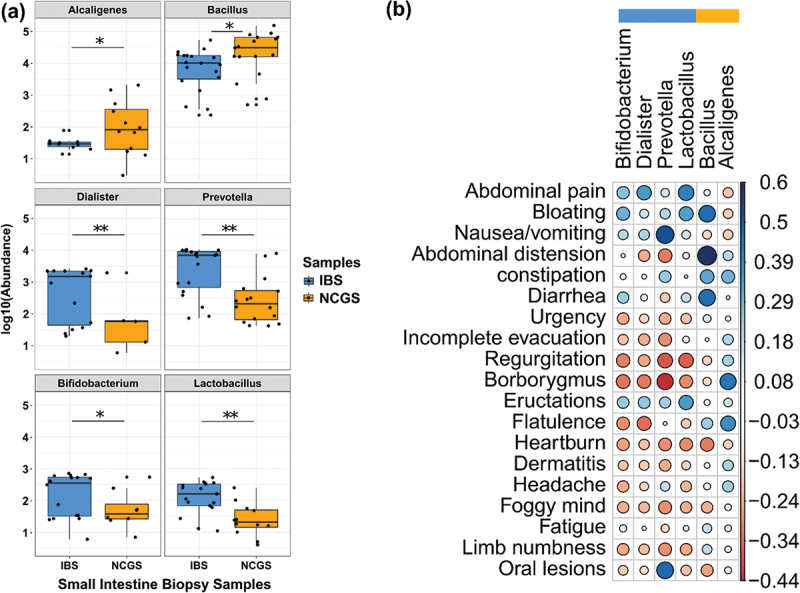
Correlation of signature microbiome in the small intestine and symptom complex in patients with NCGS. Differential signatures in the small intestine of AGA-negative IBS and NCGS patients (a). Spearman’s correlation of the signature taxa with NCGS patient symptom score at baseline (b).

The signature taxa in the small intestine of NCGS patients were further tested for any correlation with symptoms. *Bacillus* was positively correlated (Spearman’s correlation) with gastrointestinal symptoms like bloating, high stool frequency and loose stool (Figure 5a). *Alcaligenes* was found to be positively correlated with low stool frequency, incomplete evacuation, and high flatulence in patients with NCGS. Interestingly, putative beneficial taxa in small intestine of NCGS patients were found to be negatively correlated with extra-intestinal symptoms. For example, *Bifidobacterium, Prevotella, Lactobacillus* demonstrated a negative correlation with symptoms like limb numbness, joint pain, Brain fog, heartburn, borborygmus, regurgitation (Figure 5b). The differences in the microbiota between patients with NCGS and AGA-negative IBS is summarized in Table 1.

**Table 1.** Summary of the microbiota differences and signatures for patients with NCGS and IBS.

Sample Type		IBS (Disease Control)	NCGS
Small Intestinal Mucosa	Microbial Diversity	Highly significant***	
	Signatures	*Lactobacillus, Bifidobacterium, Dialister, Prevotella, Alloprevotella, Granulicatella*	*Alcaligenes, Bacillus*
Large intestinal Mucosa	Microbial Diversity	Not significant	
	Signatures	*Veillonella, Psychrobacter, Paraprevotella*	*Methanobrevibacter, Desulfovibrio, Bacillus, Rhodococcus, Achromobacter, Ochrobactrum, Acinetobacter*
Stool	Microbial Diversity	Significant*	
	Signatures	*Megasphaera, Megamonas, [Runimococcus] gnavus group*	*Elusimicrobium, Succinivibrio*

### Metagenomics reveals taxonomic and functional microbial signatures in the stool

**Figure 6.**
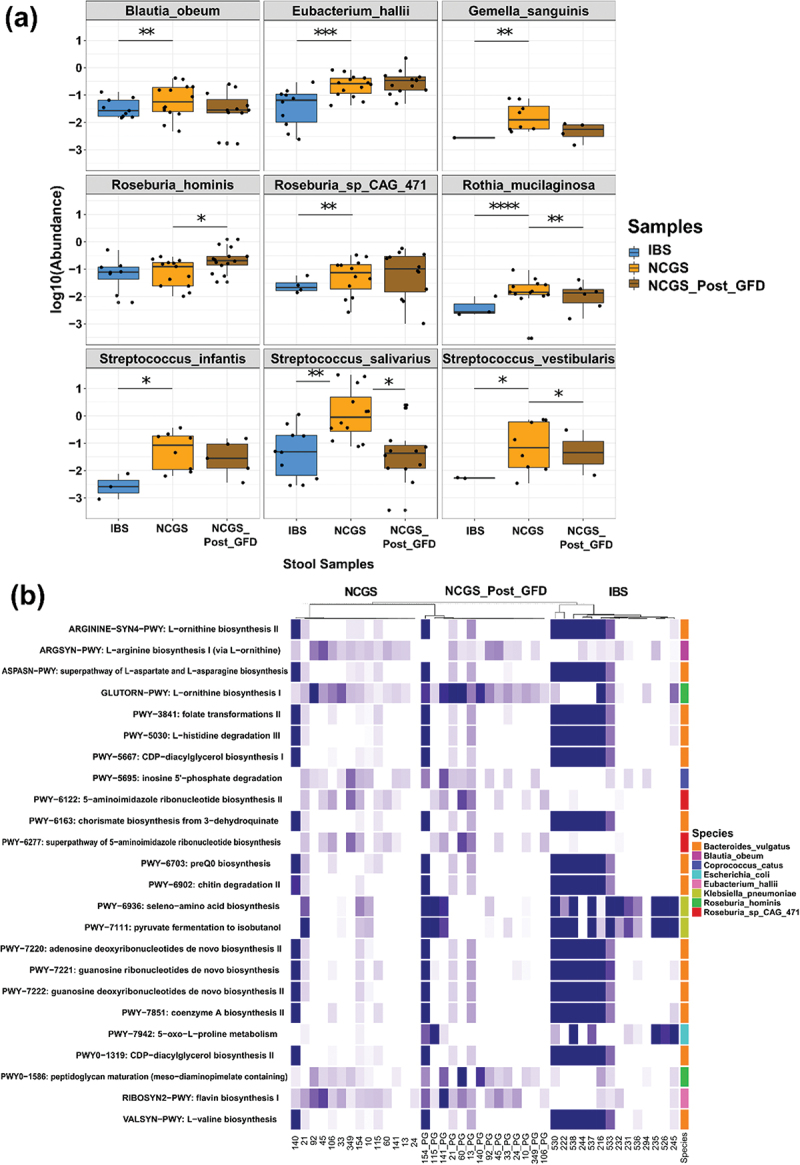
The metagenomic analysis. Differentially abundant taxa between patients with AGA-negative IBS and NCGS based on read based taxonomy analysis (a). Differential pathway abundance across the samples and taxa contributing the pathways (b).

While metabarcoding data of small intestinal samples were more reflective of the variances between groups, metagenomics data showed significant differences in the stools of patients with NCGS and AGA-negative IBS. Based on an initial screening we discovered that 12 species and 405 pathways were significantly different in patients with NCGS (pre GFD), AGA-negative IBS and NCGS (Post GFD) groups (adj. *p* < 0.05; analysis of variance [ANOVA]). Of these, 9 species and 24 pathways were found to be differentially abundant after additional Wilcoxon signed-rank test analysis for stringency [Supplementary Data 2]. Differential abundance analyses revealed that, at the species level *Blautia obeum, Eubacterium hallii, Gemella sanguinis, Rothia mucilaginosa, Streptococcus infantis, Streptococcus salivarius, Streptococcus vestibularis, Roseburia* spp CAG 471 were significantly different between NCGS and AGA-negative IBS (Figure 6a). Furthermore, we found that 19 pathways were significantly different between these two groups, where higher abundance of 16 pathways in AGA-negative IBS and enrichment of three pathways NCGS was reported. Of these, interestingly, 5-oxo-L-proline metabolism pathway of *Escherichia coli* was significantly abundant in AGA-negative IBS, whereas pathways associated with 5-aminoimidazole ribonucleotide biosynthesis in *Roseburia* spp CAG 471 and L-arginine biosynthesis in *Blautia obeum* were abundant in patients with NCGS (Figure 6b). The full list of significantly different pathways is provided in Supplementary Data 3.

### Modulation of microbiota with GFD in patients with NCGS

**Figure 7.**
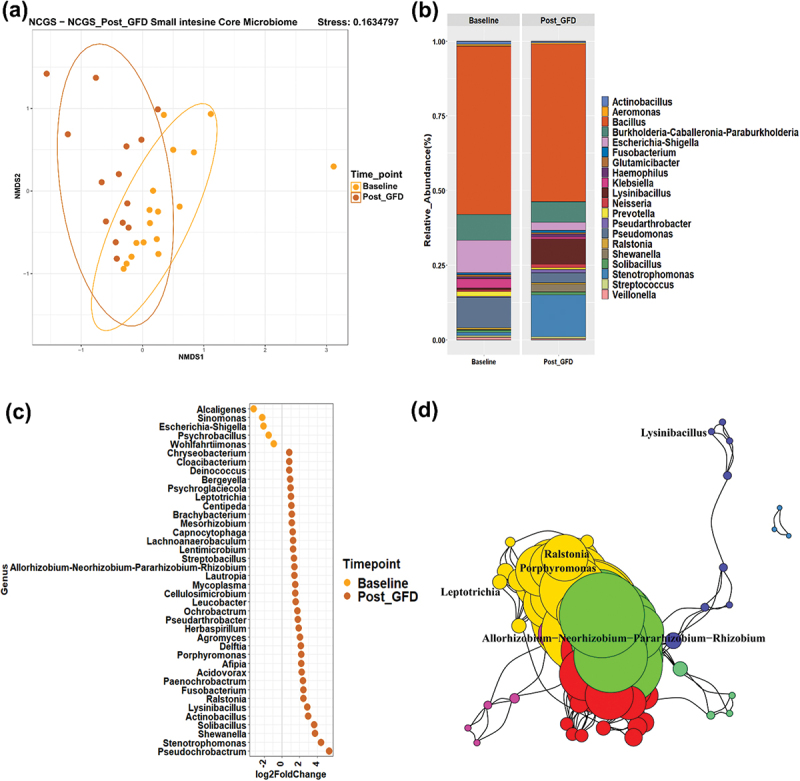
Effect of GFD on microbiota of patients with NCGS. NMDS analysis for core microbiome of the small intestine before and after GFD (a). Most abundant 20 genera associated with NCGS patients (b), differentially abundant taxa pre and post GFD (c), small intestinal signature taxa after GFD (d).

To understand the microbial changes in patients with NCGS after GFD, a comparison was done pre GFD and 6-weeks post GFD. Within this, metabarcoding analyses revealed a significant shift in the core microbiome of the small intestine with GFD in patients with NCGS (Adonis, *p* < 0.05) (Figure 7a). A decrease in the abundances of *Bacillus, Pseudomonas, Escherichia- Shigella, Klebsiella* and an increase in abundances *of Lysinibacillus, Neisseria, Stenotrophomonas* were observed in them (Figure 7b). Signature taxa for NCGS patients before GFD, i.e., *Alcaligenes and Bacillus* were observed to decrease significantly after GFD and taxa like *Solibacillus, Stenotrophomonas, Paenochrobactrum, Shewanella, Pseudochrobactrum* increased coupled with a high effect size (Figure 7c). Furthermore, co-occurrence networks revealed changes in the small intestinal mucosal signatures with GFD and *Lysinibacillus, Ralstonia, Porphyromonas, Leptotrichia, Allorhizobium,-Neorhizobium-Pararhizobium-Rhizobum* were observed as signatures (Figure 7d). On the other hand, no major significant differences were observed in microbial community composition of large intestine before and after GFD (Figure S5).

Interestingly, metagenomic analyses of stool samples identified 4 taxa to be significantly different, where *Rothia mucilaginosa, Streptococcus salivarius, Streptococcus vestibularis* were abundant in NCGS at baseline (pre-GFD), whereas *Roseburia hominis* was increased after GFD (Figure 6a). Pathway abundance analyses revealed that L-ornithine biosynthesis and peptidoglycan maturation pathways in *Roseburia hominis* were significantly higher in NCGS patients after GFD (Figure 6b).

## Discussion

In the present study, small intestinal mucosa of patients with NCGS has been observed to have higher abundances of *Bacillus, Alcaligenes, Providencia* which are known opportunists^30,31^ and lower abundances of putative beneficial bacteria. Also, the co-occurrence network-based analytics,^32^ which revealed a scattered network in the small intestine of the NCGS patients, represented by a higher modularity, larger diameter and lower overall density, in comparison to AGA-negative IBS. Altogether, these observations suggest increased dysbiosis in the small intestinal mucosa of patients with NCGS. This pattern was reversed in the large intestine and stool where most SCFA producing organisms such as *Intestinimonas, Succinivibrio* were found to be abundant in NCGS patients compared to AGA-negative IBS.^33,34^

The region-specific microbiota in the rostral to caudal axis and its importance to the host health has been highlighted previously.^35^ It has also shown that studying only stool samples for microbiome studies is insufficient and multi loci sampling is needed to obtain distinct features from the microbiome.^36^ Site-specific signatures observed in this study in the patients with NCGS and AGA-negative IBS further emphasize the necessity for comprehensive sampling for descriptive and analytic microbiome studies, especially those involving the gastrointestinal tract. Here we used DESeq2 analysis to determine the differential genera between NCGS and AGA-negative IBS patients in all three sampling sites and mapped them on bacterial co-occurrence networks to robustly extract the site-specific signatures. Incidentally, a recent report, shows that *Ruminococcus gnavus* stimulates serotonin biosynthesis in the gut and thus it may be involved in pathogenesis of IBS with diarrhea.^37^ These findings indicate robustness and reproducibility of microbiota signatures identified.

Simultaneously, correlation analysis of differential taxa in the small intestine and symptoms experienced by NCGS patients revealed that taxa abundances show a strong correlation with symptom occurrence. The findings of the present study suggest that the symptoms associated with NCGS co-occur with overrepresentation of *Alcaligenes, Bacillus* and underrepresentation of genera *Dialister, Prevotella, Bifidobacterium, Lactobacillus*; suggesting their putative role in NCGS symptoms, which we acknowledge will need targeted validation. Thomann *et al*. have recently reported strong negative correlations of *Odoribacter* and *Eubacterium* with depression and fatigue, respectively in active inflammatory bowel disease.^38^ This may explain the findings of the present study, including the association of taxa with gastrointestinal and extraintestinal symptoms, where their association with the host or their influence via metabolites could be inducing the observed symptoms. Importantly, upregulation in the majority of the amino acid biosynthesis pathways, L-ornithine biosynthesis II (ARGININE_SYN4_PWY) was observed in patients with NCGS after 6-weeks of GFD with the GLUTORN-PWY driven by *Roseburia hominis*. As reported by Tofalo *et al*, higher L-ornithine synthesis could be related to gut healing via ornithine-mediated production of polyamines which regulate epithelial apoptosis, cell division and overall gastrointestinal homeostasis.^39^ Moreover, lesser gluten degradation potential has been previously reported in patients with CeD.^40^ To the best of our knowledge, no report has recorded the gluten degradation in patients with NCGS. Interestingly, we did not find differential abundance of previously reported gluten-degrading taxa^41,42^ or pathways for gluten degradation in patients with NCGS as compared to AGA-negative IBS in the present study. In addition to bringing few facts, the data of the present study highlight the need for more stringent, site-specific, and deeper functional characterization of gut microbiota of patients with NCGS.

As the only treatment for NCGS presently is dietary elimination of gluten, i.e., GFD; which itself is likely to influence the gut microbiome and a significant change in taxa associated with starch metabolism has been demonstrated earlier as an effect of GFD.^43^ While Mazcorro *et al.*, studying the effects of GFD on NCGS patients reported a significant improvement in symptoms after 4 weeks but no significant microbiome shifts in the duodenum; they however observed an increase in population of *Pseudomonas* in patients with NCGS and CeD after GFD.^9^ In the present study, we observed a significant shift in the small intestinal mucosal core microbiome after 6 weeks of GFD, though no differences were found in *Pseudomonas* abundances (*p* > 0.05). There was, however, an increase in *Stenotrophomonas* (*p* < 0.05) in the small intestine, following GFD. Interestingly, *Pseudomonas aeruginosa* has been identified as a key species in CeD, capable of upregulating inflammatory pathways and modifying gluten degradation to generate peptides immunogenic to CeD patients.^44,45^ Whereas, *Stenotrophomonas maltophilia* previously isolated from CeD patients before and after GFD has been reported to have gluten-degradation potential.^41^

The identification of site-specific microbial signatures is particularly interesting, as they can be tested further as biomarkers for the diagnosing NCGS. The vast potential of host microbiome in development of new generation diagnostic and therapeutic aids has been established.^46^ Multiple studies have identified bacterial as well as multi kingdom signatures that can facilitate precision diagnosis of Crohn’s disease, colorectal cancer, Post-stroke cognitive impairment etc.^47–50^ Pascal et al. (2017) found 8 taxa including *Faecalibacterium, Anaerostipes, Methanobrevibacter, Collinsella*, and *Fusobacterium* that could be used for differentiation of patients with Crohn’s disease.^50^ However, Metwaly et al. (2022) discussed gut microbial signatures across inflammatory and metabolic disorders while highlighted that testing and validation of identified signatures is vital.^51^ Targeted investigation of the identified signatures along with validation in multiple NCGS cohorts will further be helpful determining true biomarkers associated with the disorder. Further, establishment of microbially-mediated pathogenesis mechanisms could help in development of secondary therapies targeted to microbial alterations such as precision probiotics.^52^

The strength of the present study is screening of a large number of patients with IBS for gluten sensitivity with the presence of AGA and making out of the diagnosis of NCGS as per Salerno’s criteria in them.^21,53^ Detection of AGA alone is not a validated biomarker for NCGS, however, results from our previous study support the exploration of AGA serology as a potential screening tool for gluten responsiveness in patients with IBS or NCGS. The study explores the effectiveness of Human Leukocyte Antigen (HLA) typing and AGA serology in the screening of IBS patients for gluten sensitivity. HLA-DQ 2/8 haplotype was found in 64.6% of AGA-positive IBS patients and in 88.2% of these patients diagnosed as NCGS. Additionally, a significant reduction in AGA levels after GFD was observed highlighting the utility of AGA as a tool to diagnose NCGS.^22^

The findings of the present study are limited due to a few factors. First, lower sample size may affect statistical power and generalizability. Additionally, only a small number of patients underwent GFD trial, and AGA-negative IBS patients were not put on GFD trial to track if they respond to intervention. Despite these limitations, we employed a stringent recruitment strategy, robust analytical techniques, and a combination of both metabarcoding and metagenomic data to identify microbiota signatures in patients with NCGS. Key aspects of this study include the characterization of multi-site and mucosa-associated microbiota and the effects of a GFD on the microbiota. The foundations established here can be further expanded with larger patient cohorts, longitudinal and long-term follow-up after GFD, more in-depth metagenomic analysis of small intestinal samples, and the incorporation of other omics methodologies, such as metabolomics, to gain deeper insights into the microbial and metabolite composition of patients with NCGS.

In summary, the mucosa-associated small intestine microbial structure is quite distinct in patients with NCGS in comparison to that with AGA-negative IBS. The study also offers insights into the small intestine as the site of pathogenesis for NCGS where the highest differences in the microbiome were observed and signatures show correlation with symptoms of NCGS patients.

## Supplementary Material

Revised_supplementary_material_ncgs_microbiota clean.docx

## Data Availability

The data generated in the study is available at the National Center for Biotechnology Information (NCBI) BioProject Repository with ID PRJNA973664 and PRJNA981497. All analysis and visualization code scripts are available at https://github.com/dixit-kunal/NCGS_IBS_microbiome_study.

## References

[1] Singh P, Arora S, Singh A, Strand TA, Makharia GK. Prevalence of celiac disease in Asia: a systematic review and meta-analysis. J Gastroenterol Hepatol. 2016 June. 31(6):1095–1101. doi:10.1111/jgh.13270. 26678020

[2] Dickerson F, Stallings C, Origoni A, Vaughan C, Khushalani S, Leister F, Yang S, Krivogorsky B, Alaedini A, Yolken R. Markers of gluten sensitivity and celiac disease in recent-onset psychosis and multi-episode schizophrenia. Biol Psychiatry. 2010 Jul. 68(1):100–104. doi:10.1016/j.biopsych.2010.03.021. 20471632

[3] Krigel A, Lebwohl B. Nonceliac gluten Sensitivity12. Adv Nutr. 2016 Nov. 7(6):1105–1110. doi:10.3945/an.116.012849. 28140327 PMC5105039

[4] Catassi C, Alaedini A, Bojarski C, Bonaz B, Bouma G, Carroccio A, Castillejo G, De Magistris L, Dieterich W, Di Liberto D, et al. The overlapping area of non-celiac gluten sensitivity (NCGS) and wheat-sensitive irritable bowel syndrome (IBS): an update. Nutrients. 2017 Nov. 9(11):1268. doi:10.3390/nu9111268. 29160841 PMC5707740

[5] Mohta S, Rajput MS, Ahuja V, Makharia GK. Emergence of celiac disease and gluten-related disorders in Asia. J Neurogastroenterol Motil. 2021 Jul. 27(3):337–346. doi:10.5056/jnm20140. 33967028 PMC8266496

[6] Transeth EL, Dale HF, Lied GA. Comparison of gut microbiota profile in celiac disease, non-celiac gluten sensitivity and irritable bowel syndrome: a systematic review. Turk J Gastroenterol. 2020 Nov. 31(11):735–745. doi:10.5152/tjg.2020.19551. 33361035 PMC7759231

[7] Barbaro MR, Cremon C, Stanghellini V, Barbara G. Recent advances in understanding non-celiac gluten sensitivity, *F1000Research*. 2018 Oct. 7:1631. doi:10.12688/f1000research.15849.1. PMC6182669 30363819

[8] Dunaevsky YE, Tereshchenkova VF, Belozersky MA, Filippova IY, Oppert B, Elpidina EN. Effective degradation of gluten and its fragments by gluten-Specific Peptidases: a review on application for the treatment of patients with gluten sensitivity. Pharmaceutics. 2021 Oct. 13(10):1603. doi:10.3390/pharmaceutics13101603. 34683896 PMC8541236

[9] Garcia-Mazcorro JF, Rivera-Gutierrez X, Cobos-Quevedo ODJ, Grube-Pagola P, Meixueiro-Daza A, Hernandez-Flores K, Cabrera-Jorge FJ, Vivanco-Cid H, Dowd SE, Remes-Troche JM. First insights into the gut microbiota of Mexican patients with celiac disease and non-celiac gluten sensitivity. Nutrients. 2018 Nov. 10(11):1641. doi:10.3390/nu10111641. 30400238 PMC6266755

[10] Dieterich W, Schuppan D, Schink M, Schwappacher R, Wirtz S, Agaimy A, Neurath MF, Zopf Y. Influence of low FODMAP and gluten-free diets on disease activity and intestinal microbiota in patients with non-celiac gluten sensitivity. Clin Nutr Edinb Scotl. 2019 Apr. 38(2):697–707. doi:10.1016/j.clnu.2018.03.017. 29653862

[11] Ahn J-S, Lkhagva E, Jung S, Kim H-J, Chung H-J, Hong S-T. Fecal microbiome does not represent whole gut microbiome. Cell Microbiol. 2023 Jan. 2023:e6868417. doi:10.1155/2023/6868417.

[12] Zhou X, Zhang S, Liu D, Qian H, Zhang D, Liu Q. The differences between fecal microbiota and intestinal fluid microbiota in colon polyps. Med (Baltim). 2021 Dec. 100(52):e28028. doi:10.1097/MD.0000000000028028. PMC8718174 34967350

[13] Constante M, Libertucci J, Galipeau HJ, Szamosi JC, Rueda G, Miranda PM, Pinto-Sanchez MI, Southward CM, Rossi L, Fontes ME, et al. Biogeographic variation and functional pathways of the gut microbiota in celiac disease. Gastroenterology. 2022 Nov. 163(5):1351–1363.e15. doi:10.1053/j.gastro.2022.06.088. 35810781

[14] Longstreth GF, Thompson WG, Chey WD, Houghton LA, Mearin F, Spiller RC. Functional bowel disorders. Gastroenterology. 2006 Apr. 130(5):1480–1491. doi:10.1053/j.gastro.2005.11.061. 16678561

[15] Sharma H, Verma AK, Das P, Dattagupta S, Ahuja V, Makharia GK. Prevalence of celiac disease in Indian patients with irritable bowel syndrome and uninvestigated dyspepsia. J Dig Dis. 2015 Aug. 16(8):443–448. doi:10.1111/1751-2980.12260. 25959064

[16] Pinto-Sanchez MI, Nardelli A, Borojevic R, De Palma G, Calo NC, McCarville J, Caminero A, Basra D, Mordhorst A, Ignatova E, et al. Gluten-free diet reduces symptoms, particularly diarrhea, in patients with irritable bowel syndrome and Antigliadin IgG. Clin Gastroenterol Hepatol Off Clin Pract J Am Gastroenterol Assoc. 2021 Nov. 19(11):2343–2352.e8. doi:10.1016/j.cgh.2020.08.040. 32827724

[17] Makharia A, Catassi C, Makharia GK. The overlap between irritable bowel syndrome and non-celiac gluten sensitivity: a clinical dilemma. Nutrients. 2015 Dec. 7(12):10417–10426. doi:10.3390/nu7125541. 26690475 PMC4690093

[18] Juge N. Relationship between mucosa-associated gut microbiota and human diseases. Biochem Soc Trans. 2022 Oct. 50(5):1225–1236. doi:10.1042/BST20201201. 36214382 PMC9704521

[19] Francis CY, Morris J, Whorwell PJ. The irritable bowel severity scoring system: a simple method of monitoring irritable bowel syndrome and its progress. Aliment Pharmacol Ther. 1997 Apr. 11(2):395–402. doi:10.1046/j.1365-2036.1997.142318000.x. 9146781

[20] Kulich KR, Madisch A, Pacini F, Piqué JM, Regula J, Van Rensburg CJ, Újszászy L, Carlsson J, Halling K, Wiklund IK. Reliability and validity of the gastrointestinal symptom rating scale (GSRS) and quality of life in reflux and dyspepsia (QOLRAD) questionnaire in dyspepsia: a six-country study. Health Qual Life Outcomes. 2008;6(1):12. doi:10.1186/1477-7525-6-12. 18237386 PMC2276197

[21] Catassi C, Elli L, Bonaz B, Bouma G, Carroccio A, Castillejo G, Cellier C, Cristofori F, De Magistris L, Dolinsek J, et al. Diagnosis of non-celiac gluten sensitivity (NCGS): the salerno experts’ criteria. Nutrients. 2015 June. 7(6):4966–4977. doi:10.3390/nu7064966. 26096570 PMC4488826

[22] Ahmed A, Dixit K, Singh A, Agarwal A, Mehtab W, Prasad S, Rajput MS, Chauhan A, Agarwal A, Mehta S, et al. Sieving out non-celiac gluten sensitivity amongst patients with irritable bowel syndrome. Dig Liver Dis. 2024 Mar. 56(3):451–457. doi:10.1016/j.dld.2023.10.014. 37985252

[23] Lim MY, Park Y-S, Kim J-H, Nam Y-D. Evaluation of fecal DNA extraction protocols for human gut microbiome studies. BMC Microbiol. 2020 Jul. 20(1):212. doi:10.1186/s12866-020-01894-5. 32680572 PMC7367376

[24] Wasimuddin KS, Ronchi SL, Leib F, Erb A, Ramette M, Ramette A. Evaluation of primer pairs for microbiome profiling from soils to humans within the one health framework. Mol Ecol Resour. 2020 Nov. 20(6):1558–1571. doi:10.1111/1755-0998.13215. 32599660 PMC7693082

[25] Callahan BJ, McMurdie PJ, Rosen MJ, Han AW, Johnson AJA, Holmes SP. DADA2: high-resolution sample inference from illumina amplicon data. Nat Methods. 2016 Jul. 13(7):581–583. doi:10.1038/nmeth.3869. 27214047 PMC4927377

[26] Quast C, Pruesse E, Yilmaz P, Gerken J, Schweer T, Yarza P, Peplies J, Glöckner FO. The SILVA ribosomal RNA gene database project: improved data processing and web-based tools. Nucleic Acids Res. 2013 Jan. 41(Database issue):D590–D596. doi:10.1093/nar/gks1219. 23193283 PMC3531112

[27] Love MI, Huber W, Anders S. Moderated estimation of fold change and dispersion for rna-seq data with DESeq2. Genome Biol. 2014 Dec. 15(12):550. doi:10.1186/s13059-014-0550-8. 25516281 PMC4302049

[28] “Integrating taxonomic, functional, and strain-level profiling of diverse microbial communities with bioBakery 3 | eLife. [Accessed 2023 May 28. [Online]. Available: https://elifesciences.org/articles/65088. 10.7554/eLife.65088 PMC8096432 33944776

[29] Nogal A, Valdes AM, Menni C. The role of short-chain fatty acids in the interplay between gut microbiota and diet in cardio-metabolic health. Gut Microbes. 2021;13(1):1897212. doi:10.1080/19490976.2021.1897212. 33764858 PMC8007165

[30] Shah MM, Odoyo E, Ichinose Y. Epidemiology and pathogenesis of providencia alcalifaciens infections. Am J Trop Med Hyg. 2019 Aug. 101(2):290–293. doi:10.4269/ajtmh.18-0376. 31218997 PMC6685554

[31] de Blackburn CW, McClure PJ. 24 - pathogenic bacillus species. In: Blackburn CDW McClure PJ, editors, in Woodhead Publishing Series in Food Science, Foodborne pathogens. Second Edition) ed. Technology and Nutrition. Woodhead Publishing; 2009. 844–888. doi:10.1533/9781845696337.2.844.

[32] Hall CV, Lord A, Betzel R, Zakrzewski M, Simms LA, Zalesky A, Radford-Smith G, Cocchi L. Co-existence of network architectures supporting the human gut microbiome. iScience. 2019 Dec. 22:380–391. doi:10.1016/j.isci.2019.11.032. 31812808 PMC6911941

[33] Salamone D, Rivellese AA, Vetrani C. The relationship between gut microbiota, short-chain fatty acids and type 2 diabetes mellitus: the possible role of dietary fibre. Acta Diabetol. 2021;58(9):1131–1138. doi:10.1007/s00592-021-01727-5. 33970303 PMC8316221

[34] Fernández-Veledo S, Vendrell J. Gut microbiota-derived succinate: friend or foe in human metabolic diseases? Rev Endocr Metab Disord. 2019;20(4):439–447. doi:10.1007/s11154-019-09513-z. 31654259 PMC6938788

[35] Martinez-Guryn K, Leone V, Chang EB. Regional diversity of the gastrointestinal microbiome. Cell Host & Microbe. 2019 Sep. 26(3):314–324. doi:10.1016/j.chom.2019.08.011. 31513770 PMC6750279

[36] Levitan O, Ma L, Giovannelli D, Burleson DB, McCaffrey P, Vala A, Johnson DA. The gut microbiome–does stool represent right? Heliyon. 2023 Mar. 9(3):e13602. doi:10.1016/j.heliyon.2023.e13602. 37101508 PMC10123208

[37] Zhai L, Huang C, Ning Z, Zhang Y, Zhuang M, Yang W, Wang X, Wang J, Zhang L, Xiao H, et al. Ruminococcus gnavus plays a pathogenic role in diarrhea-predominant irritable bowel syndrome by increasing serotonin biosynthesis. Cell Host & Microbe. 2023 Jan. 31(1):33–44.e5. doi:10.1016/j.chom.2022.11.006. 36495868

[38] Thomann AK, Wüstenberg T, Wirbel J, Knoedler L-L, Thomann PA, Zeller G, Ebert MP, Lis S, Reindl W. Depression and fatigue in active IBD from a microbiome perspective—a Bayesian approach to faecal metagenomics. BMC Med. 2022 Oct. 20(1):366. doi:10.1186/s12916-022-02550-7. 36244970 PMC9575298

[39] Tofalo R, Cocchi S, Suzzi G. Polyamines and gut microbiota. Front Nutr. 2019 Feb. 6:16. doi:10.3389/fnut.2019.00016. 30859104 PMC6397830

[40] Bodkhe R, Shetty SA, Dhotre DP, Verma AK, Bhatia K, Mishra A, Kaur G, Pande P, Bangarusamy DK, Santosh BP, et al. Comparison of Small Gut and whole Gut microbiota of first-degree relatives with adult celiac disease patients and controls. Front Microbiol. 2019 Feb. 10:164. doi:10.3389/fmicb.2019.00164. 30800106 PMC6376745

[41] Herrán AR, Pérez-Andrés J, Caminero A, Nistal E, Vivas S, Ruiz de Morales JM, Casqueiro J. Gluten-degrading bacteria are present in the human small intestine of healthy volunteers and celiac patients. Res Microbiol. 2017 Sep. 168(7):673–684. doi:10.1016/j.resmic.2017.04.008. 28526528

[42] Kõiv V, Tenson T. Gluten-degrading bacteria: availability and applications. Appl Microbiol Biotechnol. 2021 Apr. 105(8):3045–3059. doi:10.1007/s00253-021-11263-5. 33837830 PMC8053163

[43] Bonder MJ, Tigchelaar EF, Cai X, Trynka G, Cenit MC, Hrdlickova B, Zhong H, Vatanen, T, et al. The influence of a short-term gluten-free diet on the human gut microbiome - PubMed Genome Med. [Accessed 2023 May 28 2016 doi:10.1186/s13073-016-0295-y. [Online]. Available: https://pubmed.ncbi.nlm.nih.gov/27102333/. PMC4841035 27102333

[44] Caminero A, McCarville JL, Galipeau HJ, Deraison C, Bernier SP, Constante M, Rolland C, Meisel M, Murray JA, Yu XB, et al. Duodenal bacterial proteolytic activity determines sensitivity to dietary antigen through protease-activated receptor-2. Nat Commun. 2019 Mar. 10(1):1198. doi:10.1038/s41467-019-09037-9. 30867416 PMC6416356

[45] Caminero A, Galipeau HJ, McCarville JL, Johnston CW, Bernier SP, Russell AK, Jury J, Herran AR, Casqueiro J, Tye-Din JA, et al. Duodenal bacteria from patients with celiac disease and healthy subjects distinctly affect gluten breakdown and immunogenicity. Gastroenterology. 2016 Oct. 151(4):670–683. doi:10.1053/j.gastro.2016.06.041. 27373514

[46] Hajjo R, Sabbah DA, Al Bawab AQ. Unlocking the potential of the human microbiome for identifying disease diagnostic biomarkers. Diagnostics. 2022 Jul. 12(7):1742. doi:10.3390/diagnostics12071742. 35885645 PMC9315466

[47] Ling Y, Gong T, Zhang J, Gu Q, Gao X, Weng X, Liu J, Sun J. Gut microbiome signatures are biomarkers for cognitive impairment in patients with ischemic stroke. Front Aging Neurosci. 2020 Oct. 12:511562. doi:10.3389/fnagi.2020.511562. 33192448 PMC7645221

[48] Zhu X, Xu P, Zhu R, Gao W, Yin W, Lan P, Zhu L, Jiao N. Multi-kingdom microbial signatures in excess body weight colorectal cancer based on global metagenomic analysis. Commun Biol. 2024 Jan. 7(1):1–14. doi:10.1038/s42003-023-05714-0. 38182885 PMC10770074

[49] Oliver L, Camps B, Julià-Bergkvist D, Amoedo J, Ramió-Pujol S, Malagón M, Bahí A, Torres P, Domènech E, Guardiola J, et al. Definition of a microbial signature as a predictor of endoscopic post-surgical recurrence in patients with Crohn’s disease. Front Mol Med. 2023 Feb. 3. doi:10.3389/fmmed.2023.1046414. PMC11285546 39086694

[50] Pascal V, Pozuelo M, Borruel N, Casellas F, Campos D, Santiago A, Martinez X, Varela E, Sarrabayrouse G, Machiels K, et al. A microbial signature for Crohn’s disease. Gut. 2017 May. 66(5):813–822. doi:10.1136/gutjnl-2016-313235. 28179361 PMC5531220

[51] Metwaly A, Reitmeier S, Haller D. Microbiome risk profiles as biomarkers for inflammatory and metabolic disorders. Nat Rev Gastroenterol Hepatol. 2022 June. 19(6):383–397. doi:10.1038/s41575-022-00581-2. 35190727

[52] Galipeau HJ, Verdu EF. The double-edged sword of gut bacteria in celiac disease and implications for therapeutic potential. Mucosal Immunol. 2022 Feb. 15(2):235–243. doi:10.1038/s41385-021-00479-3. 35031683

[53] Infantino M, Meacci F, Grossi V, Macchia D, Manfredi M. Anti-gliadin antibodies in non-celiac gluten sensitivity. Minerva Gastroenterol Dietol. 2017 Mar. 63(1):1–4. doi:10.23736/S1121-421X.16.02351-5. 27845509

