# Supplementary material for: Site-Specific Gut Microbial Signatures in Non-Celiac Gluten Sensitivity
Source: Gut Microbes Rep. 2024 Dec 18;1(1):2438621. doi: 10.1080/29933935.2024.2438621 (PMC12940131; doi:10.1080/29933935.2024.2438621)
Supplement: Revised_supplementary_material_ncgs_microbiota clean.docx [file KGMR_A_2438621_SM1741.docx]

**Site-specific microbial signatures in non-celiac gluten sensitivity**

Kunal Dixit^1^*, Anam Ahmed^2^*, Alka Singh^2^, Mitali Inamdar^3^, Sonal Chavan^3^, Rahul Bodkhe^4^, Wajiha Mehtab^5^, Ashish Chauhan^2^, Sunil D. Saroj^1^, Vineet Ahuja^2^, Yogesh Shouche^3,6^, Dhiraj Dhotre^3#^, Govind Makaharia^2#^

**Affiliation:**

^1^Symbiosis School of Biological Sciences, Symbiosis International (Deemed University) Pune, India

^2^Department of Gastroenterology and Human nutrition, All India Institute of Medical Sciences, India

^3^National Centre for Cell Science, Pune, India

^4^University of California, Berkeley, USA

^5^Department of Home Science, University of Delhi, New Delhi, India

^6^SKAN Research Trust, Bengaluru, India

**Supplementary material:**

1. **Supplementary Data 1: Co-occurrence co-exclusion network analysis statistics**

|  | **Stool** | | | | **Small intestinal** | | | **Large intestinal** | | |
| --- | --- | --- | --- | --- | --- | --- | --- | --- | --- | --- |
| **Stats** | **Healthy** | **AGA negative IBS** | **NCGS** | **NCGS_PG** | **AGA negative IBS** | **NCGS** | **NCGS_PG** | **AGA negative IBS** | **NCGS** | **NCGS_PG** |
| **Relative abundance threshold (%)** | 0.2 | 0.2 | 0.2 | 0.5 | 0.5 | 0.5 | 0.5 | 0.5 | 0.5 | 0.5 |
| **Nodes** | 34 | 41 | 39 | 49 | 92 | 57 | 116 | 86 | 73 | 82 |
| **Edges** | 134 | 75 | 113 | 161 | 1093 | 284 | 1647 | 507 | 577 | 1068 |
| **Density** | 0.239 | 0.091 | 0.152 | 0.137 | 0.261 | 0.178 | 0.247 | 0.139 | 0.220 | 0.322 |
| **Transitivity** | 0.558 | 0.667 | 0.713 | 0.686 | 0.910 | 0.730 | 0.713 | 0.664 | 0.662 | 0.742 |
| **Avg_transitivity** | 0.529 | 0.669 | 0.635 | 0.621 | 0.873 | 0.761 | 0.757 | 0.695 | 0.729 | 0.780 |
| **Modularity** | 0.340 | 0.621 | 0.415 | 0.512 | 0.460 | 0.563 | 0.370 | 0.563 | 0.384 | 0.218 |
| **Diameter** | 1.864 | 4.591 | 4.957 | 4.444 | 2.968 | 3.541 | 4.546 | 4.604 | 4.343 | 3.994 |
| **Assortativity** | 0.189 | 0.711 | 0.573 | 0.562 | 0.861 | 0.338 | 0.345 | 0.569 | 0.492 | 0.309 |
| **Mean_distance** | 0.804 | 1.955 | 1.834 | 1.811 | 1.435 | 1.565 | 1.414 | 1.778 | 1.596 | 1.266 |
| **Cliques** | 42 | 24 | 27 | 38 | 44 | 31 | 270 | 68 | 117 | 120 |
| **Clusters** | 4 | 9 | 8 | 5 | 4 | 5 | 5 | 6 | 4 | 5 |

1. **Supplementary Data 2: Differentially abundant taxa between study groups (AGA negative IBS, NCGS and NCGS post GFD) based on shotgun metagenomics**

| Sr. No. | Species | group1 | group2 | p.format | p.signif | method |
| --- | --- | --- | --- | --- | --- | --- |
| 1 | *Rothia_mucilaginosa* | ibs | ncgs | 4.74E-05 | **** | Wilcoxon |
| 2 | *Rothia_mucilaginosa* | ncgs | pg_ncgs | 0.009513 | ** | Wilcoxon |
| 3 | *Gemella_sanguinis* | ibs | ncgs | 0.003635 | ** | Wilcoxon |
| 4 | *Streptococcus_infantis* | ibs | ncgs | 0.017682 | * | Wilcoxon |
| 5 | *Streptococcus_salivarius* | ibs | ncgs | 0.009092 | ** | Wilcoxon |
| 6 | *Streptococcus_salivarius* | ncgs | pg_ncgs | 0.015711 | * | Wilcoxon |
| 7 | *Streptococcus_vestibularis* | ibs | ncgs | 0.010895 | * | Wilcoxon |
| 8 | *Streptococcus_vestibularis* | ncgs | pg_ncgs | 0.019719 | * | Wilcoxon |
| 9 | *Eubacterium_hallii* | ibs | ncgs | 0.00072 | *** | Wilcoxon |
| 10 | *Eubacterium_hallii* | ibs | pg_ncgs | 0.000837 | *** | Wilcoxon |
| 11 | *Blautia_obeum* | ibs | ncgs | 0.009236 | ** | Wilcoxon |
| 12 | *Roseburia_hominis* | ibs | pg_ncgs | 0.000963 | *** | Wilcoxon |
| 13 | *Roseburia_hominis* | ncgs | pg_ncgs | 0.0106 | * | Wilcoxon |
| 14 | *Roseburia_sp_CAG_471* | ibs | ncgs | 0.002373 | ** | Wilcoxon |
| 15 | *Roseburia_sp_CAG_471* | ibs | pg_ncgs | 0.00172 | ** | Wilcoxon |

1. **Supplementary Data 3: Differentially abundant Pathways and contributing taxa between study groups (AGA negative IBS, NCGS and NCGS post GFD) based on shotgun metagenomics**

| Sr. No. | Pathway | Group1 | Group2 | p.format | p.signif | method |
| --- | --- | --- | --- | --- | --- | --- |
| 1 | PWY-7221: guanosine ribonucleotides de novo biosynthesis\|g__Bacteroides.s__Bacteroides_vulgatus | IBS | NCGS_PG | 0.0496 | * | Wilcoxon |
| 2 | VALSYN-PWY: L-valine biosynthesis\|g__Bacteroides.s__Bacteroides_vulgatus | IBS | NCGS | 0.0176 | * | Wilcoxon |
| 3 | PWY-6163: chorismate biosynthesis from 3-dehydroquinate\|g__Bacteroides.s__Bacteroides_vulgatus | IBS | NCGS | 0.0242 | * | Wilcoxon |
| 4 | GLUTORN-PWY: L-ornithine biosynthesis I\|g__Roseburia.s__Roseburia_hominis | NCGS | NCGS_PG | 0.0332 | * | Wilcoxon |
| 5 | PWY-7111: pyruvate fermentation to isobutanol (engineered)\|g__Klebsiella.s__Klebsiella_pneumoniae | IBS | NCGS | 0.0418 | * | Wilcoxon |
| 6 | ARGSYN-PWY: L-arginine biosynthesis I (via L-ornithine)\|g__Blautia.s__Blautia_obeum | IBS | NCGS | 0.0055 | ** | Wilcoxon |
| 7 | PWY0-1586: peptidoglycan maturation (meso-diaminopimelate containing)\|g__Roseburia.s__Roseburia_hominis | IBS | NCGS_PG | 0.042 | * | Wilcoxon |
| 8 | PWY0-1586: peptidoglycan maturation (meso-diaminopimelate containing)\|g__Roseburia.s__Roseburia_hominis | NCGS | NCGS_PG | 0.0142 | * | Wilcoxon |
| 9 | PWY-3841: folate transformations II (plants)\|g__Bacteroides.s__Bacteroides_vulgatus | IBS | NCGS | 0.0127 | * | Wilcoxon |
| 10 | PWY-7851: coenzyme A biosynthesis II (eukaryotic)\|g__Bacteroides.s__Bacteroides_vulgatus | IBS | NCGS | 0.0283 | * | Wilcoxon |
| 11 | PWY-6936: seleno-amino acid biosynthesis (plants)\|g__Klebsiella.s__Klebsiella_pneumoniae | IBS | NCGS | 0.0297 | * | Wilcoxon |
| 12 | PWY0-1319: CDP-diacylglycerol biosynthesis II\|g__Bacteroides.s__Bacteroides_vulgatus | IBS | NCGS | 0.0101 | * | Wilcoxon |
| 13 | PWY-5667: CDP-diacylglycerol biosynthesis I\|g__Bacteroides.s__Bacteroides_vulgatus | IBS | NCGS | 0.0101 | * | Wilcoxon |
| 14 | RIBOSYN2-PWY: flavin biosynthesis I (bacteria and plants)\|g__Eubacterium.s__Eubacterium_hallii | IBS | NCGS_PG | 0.0396 | * | Wilcoxon |
| 15 | ASPASN-PWY: superpathway of L-aspartate and L-asparagine biosynthesis\|g__Bacteroides.s__Bacteroides_vulgatus | IBS | NCGS | 0.0293 | * | Wilcoxon |
| 16 | PWY-6703: preQ0 biosynthesis\|g__Bacteroides.s__Bacteroides_vulgatus | IBS | NCGS | 0.0101 | * | Wilcoxon |
| 17 | PWY-7220: adenosine deoxyribonucleotides de novo biosynthesis II\|g__Bacteroides.s__Bacteroides_vulgatus | IBS | NCGS | 0.0242 | * | Wilcoxon |
| 18 | PWY-7222: guanosine deoxyribonucleotides de novo biosynthesis II\|g__Bacteroides.s__Bacteroides_vulgatus | IBS | NCGS | 0.0242 | * | Wilcoxon |
| 19 | PWY-5030: L-histidine degradation III\|g__Bacteroides.s__Bacteroides_vulgatus | IBS | NCGS | 0.0485 | * | Wilcoxon |
| 20 | ARGININE-SYN4-PWY: L-ornithine biosynthesis II\|g__Bacteroides.s__Bacteroides_vulgatus | IBS | NCGS | 0.0295 | * | Wilcoxon |
| 21 | PWY-6902: chitin degradation II (Vibrio)\|g__Bacteroides.s__Bacteroides_vulgatus | IBS | NCGS | 0.0333 | * | Wilcoxon |
| 22 | PWY-5695: inosine 5'-phosphate degradation\|g__Coprococcus.s__Coprococcus_catus | IBS | NCGS_PG | 0.0121 | * | Wilcoxon |
| 23 | PWY-7942: 5-oxo-L-proline metabolism\|g__Escherichia.s__Escherichia_coli | IBS | NCGS | 0.0444 | * | Wilcoxon |
| 24 | PWY-6122: 5-aminoimidazole ribonucleotide biosynthesis II\|g__Roseburia.s__Roseburia_sp_CAG_471 | IBS | NCGS | 0.0444 | * | Wilcoxon |
| 25 | PWY-6277: superpathway of 5-aminoimidazole ribonucleotide biosynthesis\|g__Roseburia.s__Roseburia_sp_CAG_471 | IBS | NCGS | 0.0444 | * | Wilcoxon |

**Supplementary Figures**

Figure S1


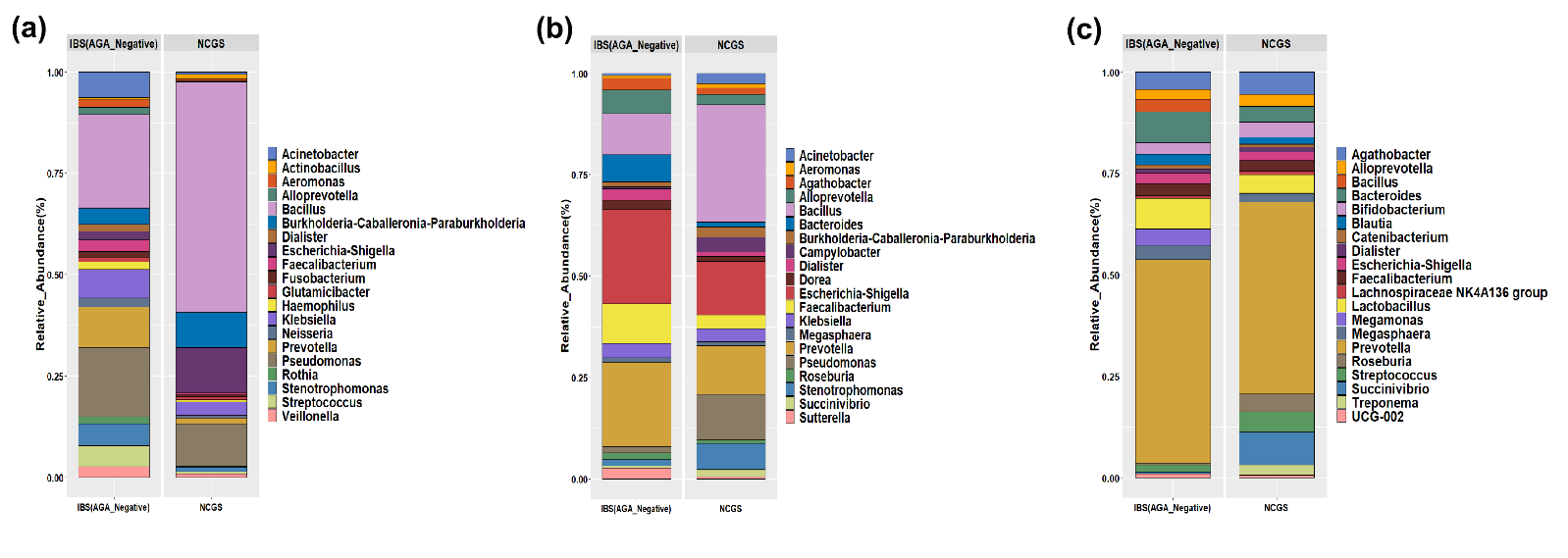


**Figure S1:** Microbial diversity differences between patients. Top 20 most abundant bacterial genera observed in small intestine (a), large intestine (b), and stool samples (c) of patients with NCGS and AGA negative IBS

Figure S2


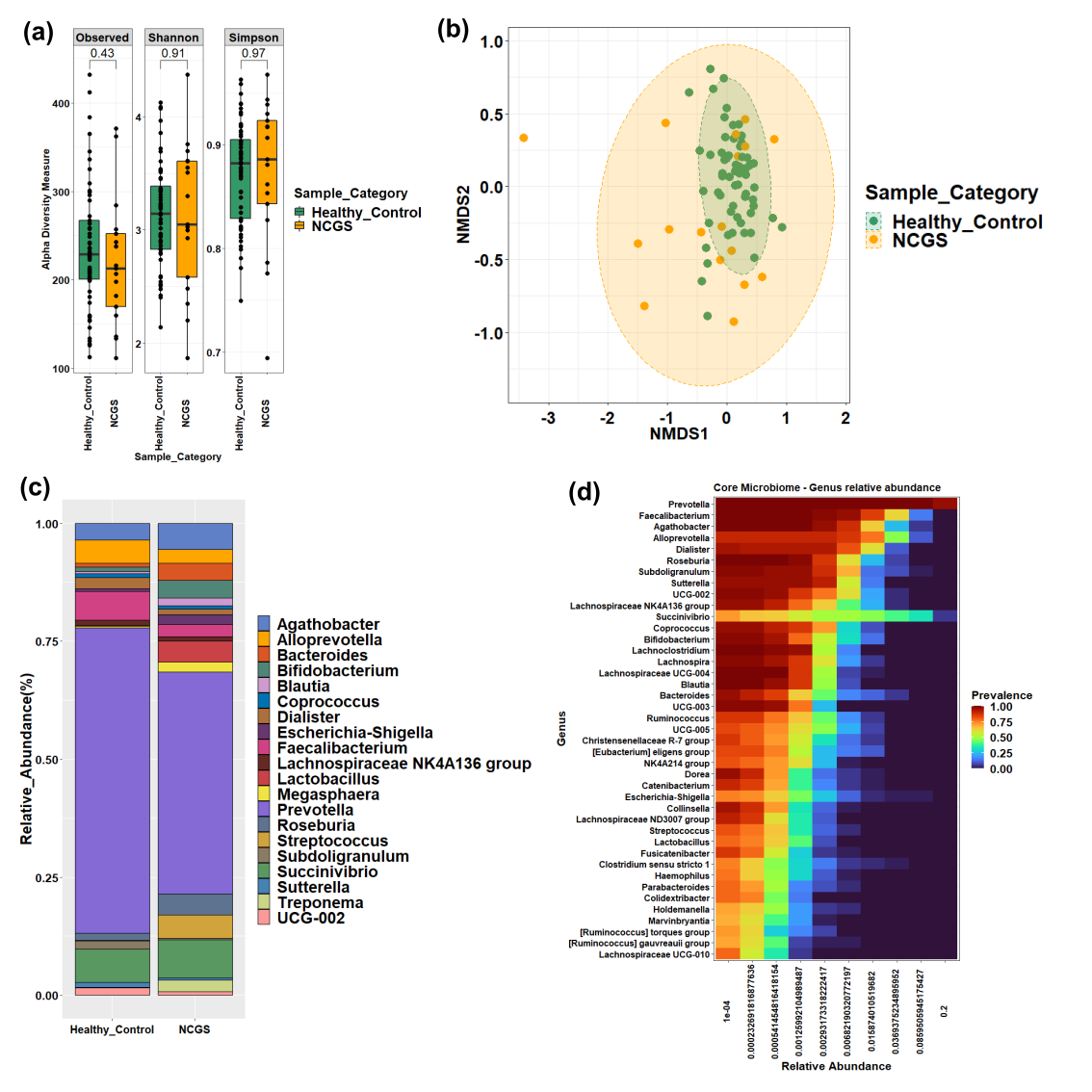


**Figure S2:** Comparison of NCGS patients stool microbiota with healthy stool microbial diversity. Alpha diversity with no significant difference between microbiota (a), NMDS analysis showing high sample to sample variation in NCGS patients compared to healthy controls (b), bacterial diversity of top 20 genera associated with the two conditions (c), Core microbiome of healthy controls (d).

Figure S3


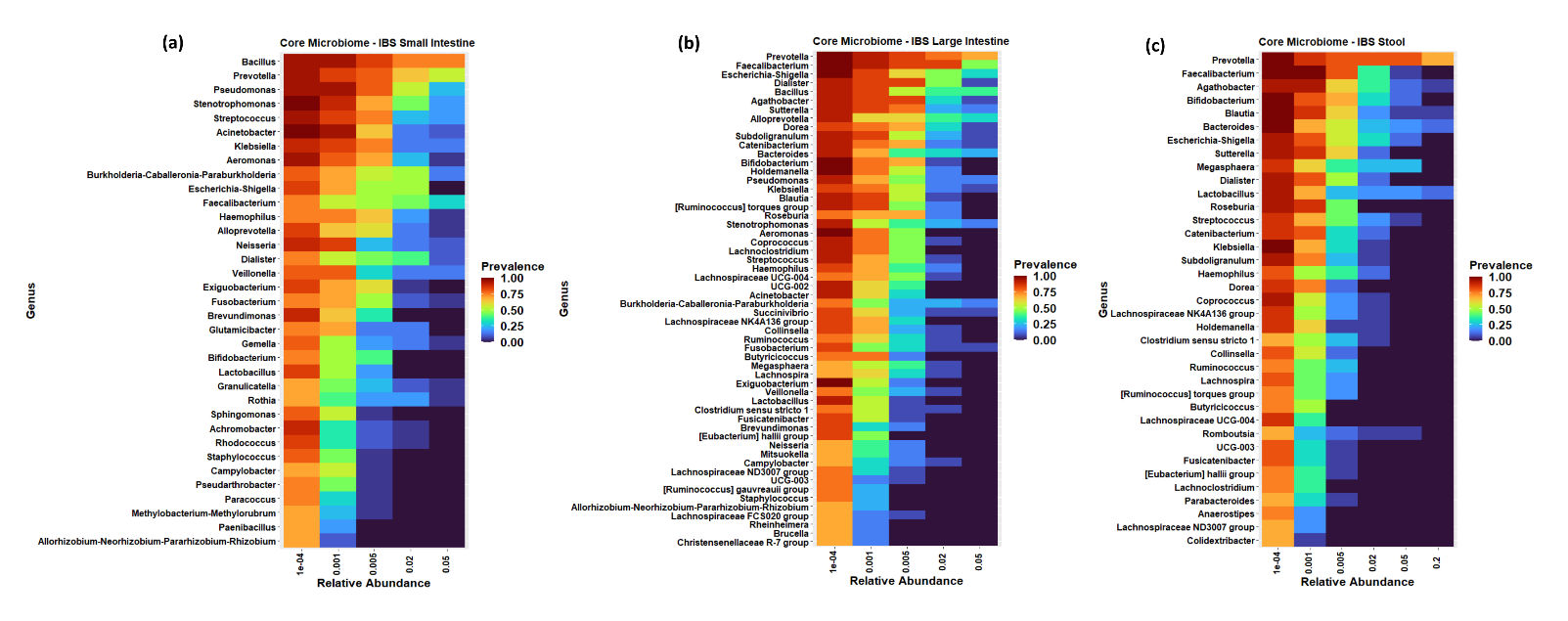


**Figure S3:** Core microbiome of patients with AGA negative IBS in the small intestine (a), large intestine (b) and stool samples (c).

Figure S4


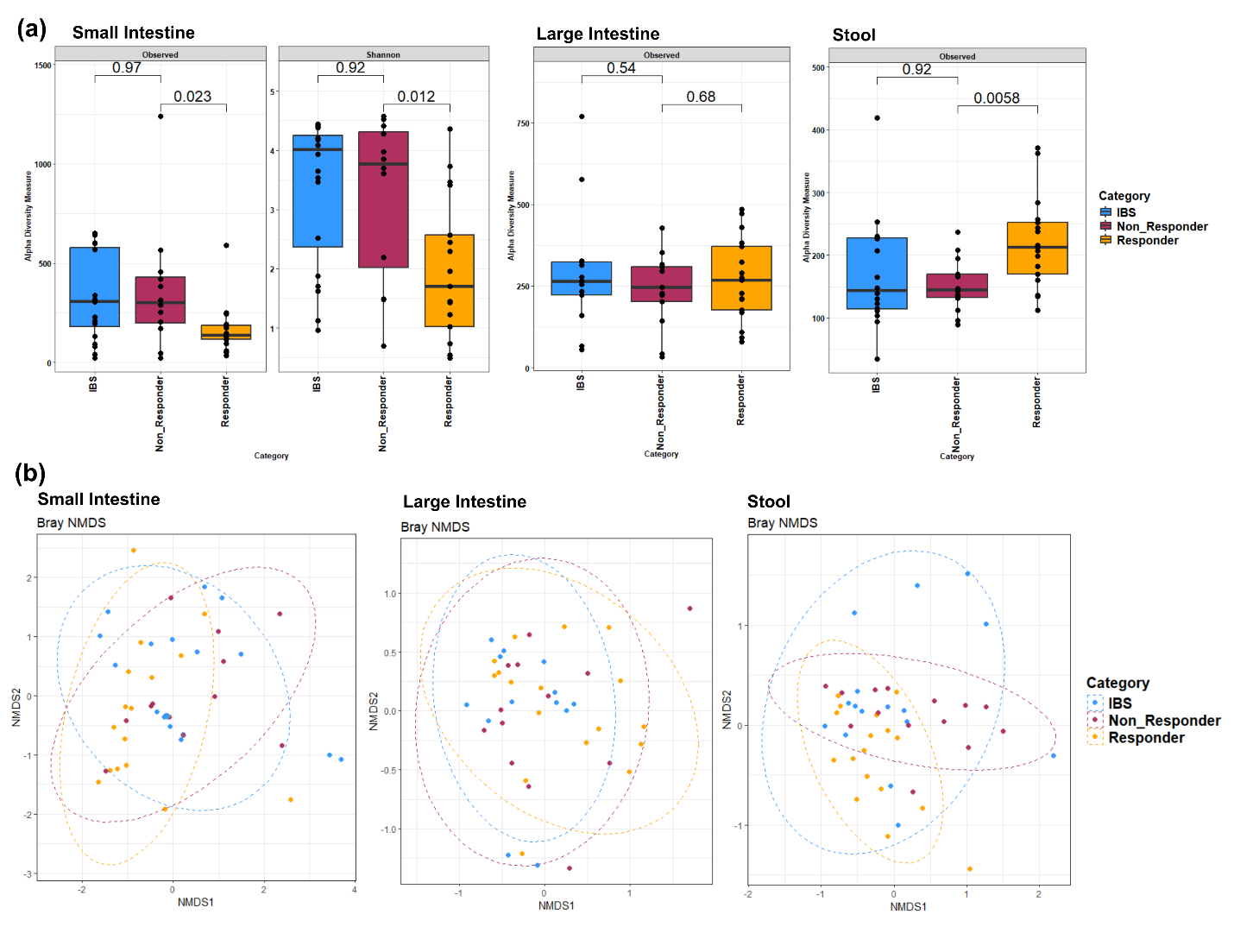


**Figure S4:** Microbial diversity associated with non-responders to GFD (AGA positive IBS patients) is similar to AGA negative IBS patients. Alpha diversity (a), beta diversity (b) compares AGA negative IBS patients, non-responders to GFD, and responders to GFD (NCGS patients).

Figure S5


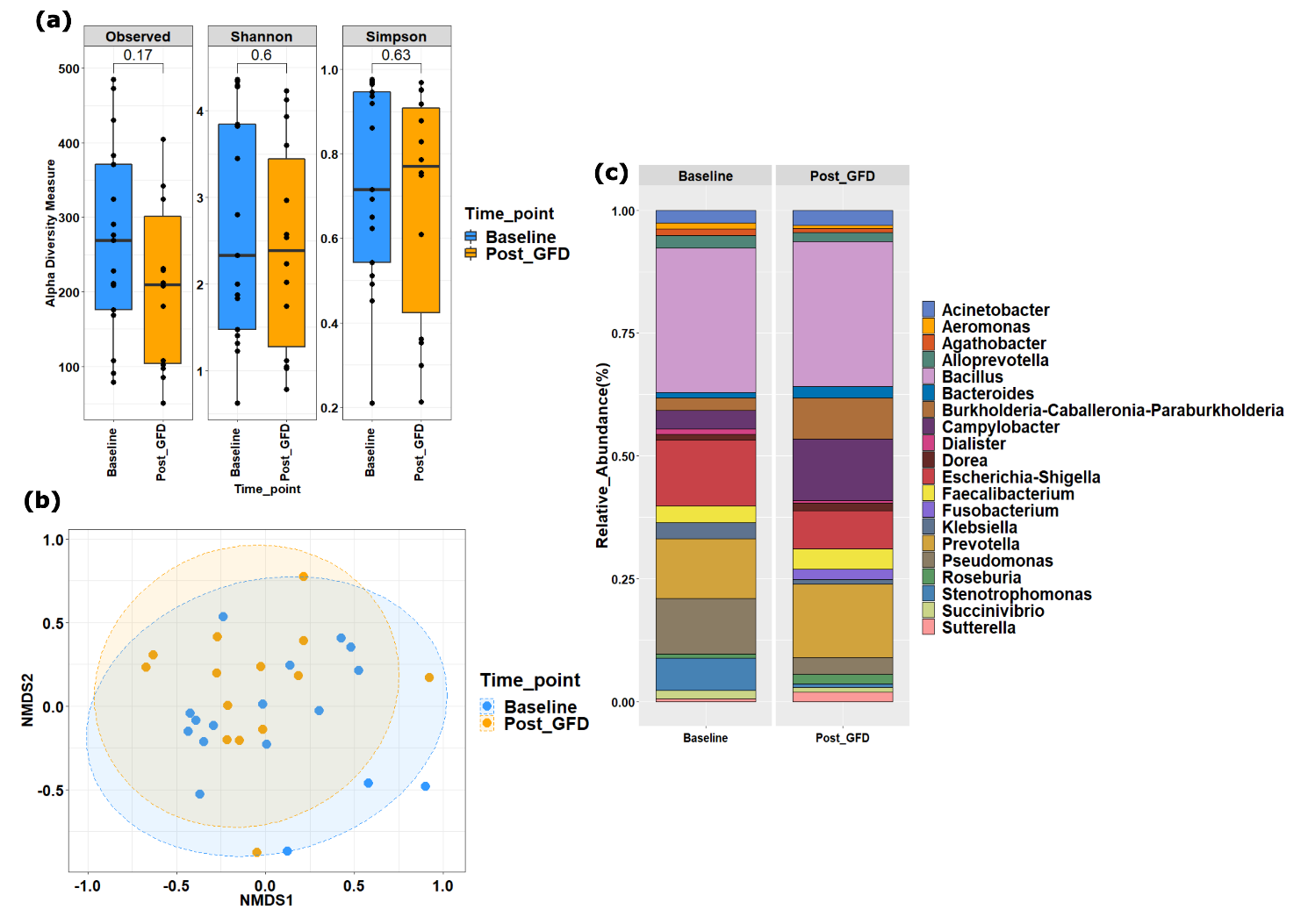


**Figure S5:** Microbial diversity associated with large intestinal biopsy samples of patients with NCGS before and after GFD. No significant differences between alpha diversity measures were found (a), NMDS plot showing overlapping microbial communities after intervention (b), highly abundant genera associated with NCGS patients pre and post GFD (c).
